# Cancer Stem Cells as Central Nodes in Therapeutic Strategies

**DOI:** 10.3390/cells15211978

**Published:** 2026-10-30

**Authors:** Saima Usman, William Andrew Yeudall, Ahmad Jamal, Muy-Teck Teh, Ahmad Waseem

**Affiliations:** 1 Department of Oral Pathology, National University of Medical Sciences NUMS, Rawalpindi 46000, Pakistan; 2 Department of Oral Biology, The Dental College of Georgia, Augusta University, Augusta, GA 30912, USA; 3 Georgia Cancer Center, Augusta University, Augusta, GA 30912, USA; 4 Clinical Biochemistry Department, Faculty of Medicine, King Abdulaziz University, Jeddah 21589, Saudi Arabia; 5 Centre for Oral Immunobiology and Regenerative Medicine, Institute of Dentistry, Faculty of Medicine and Dentistry, Queen Mary University of London, Turner Street, London E1 2AD, UK

**Keywords:** metastasis, stem cells, epithelial mesenchymal transition, cancer progression, tumour microenvironment

## Abstract

Cancer stem cells (CSCs) are a small population of cancer cells that endow unique traits of self-renewal, differentiation, tumorigenicity, quiescence and therapeutic resistance, ultimately leading to cancer relapse and poor prognosis. Conventional chemo- and radiotherapies effectively destroy rapidly dividing cancer cells; however, CSCs are largely refractory to these treatments due to special characteristics that include metabolic plasticity, low reactive oxygen species (ROS) levels, apoptosis inhibition and modified DNA repair systems, effectively hijacking the tumour microenvironment and evading host immune surveillance. This review summarises the potential CSC diagnostic methods that can be used in routine clinical investigation for tailoring personalised treatment plans both pre- and post-surgery. In this way, a CSC gene signature, reactivity and load can be estimated and, by using artificial intelligence and machine learning algorithms in a personalised therapeutic regimen, the most effective treatment response can be predicted. Furthermore, we propose incorporation of CSC-targeted therapies in conjugation with conventional chemo- and radiotherapies to eradicate CSCs, thereby eliminating the risk of relapse and therapy failure in cancer patients. This review also describes multiple potential molecular targets that may be used to target CSCs and emphasises the need for further evaluation, in order that they may be applied clinically.

## 1. Introduction

**Figure 1:**
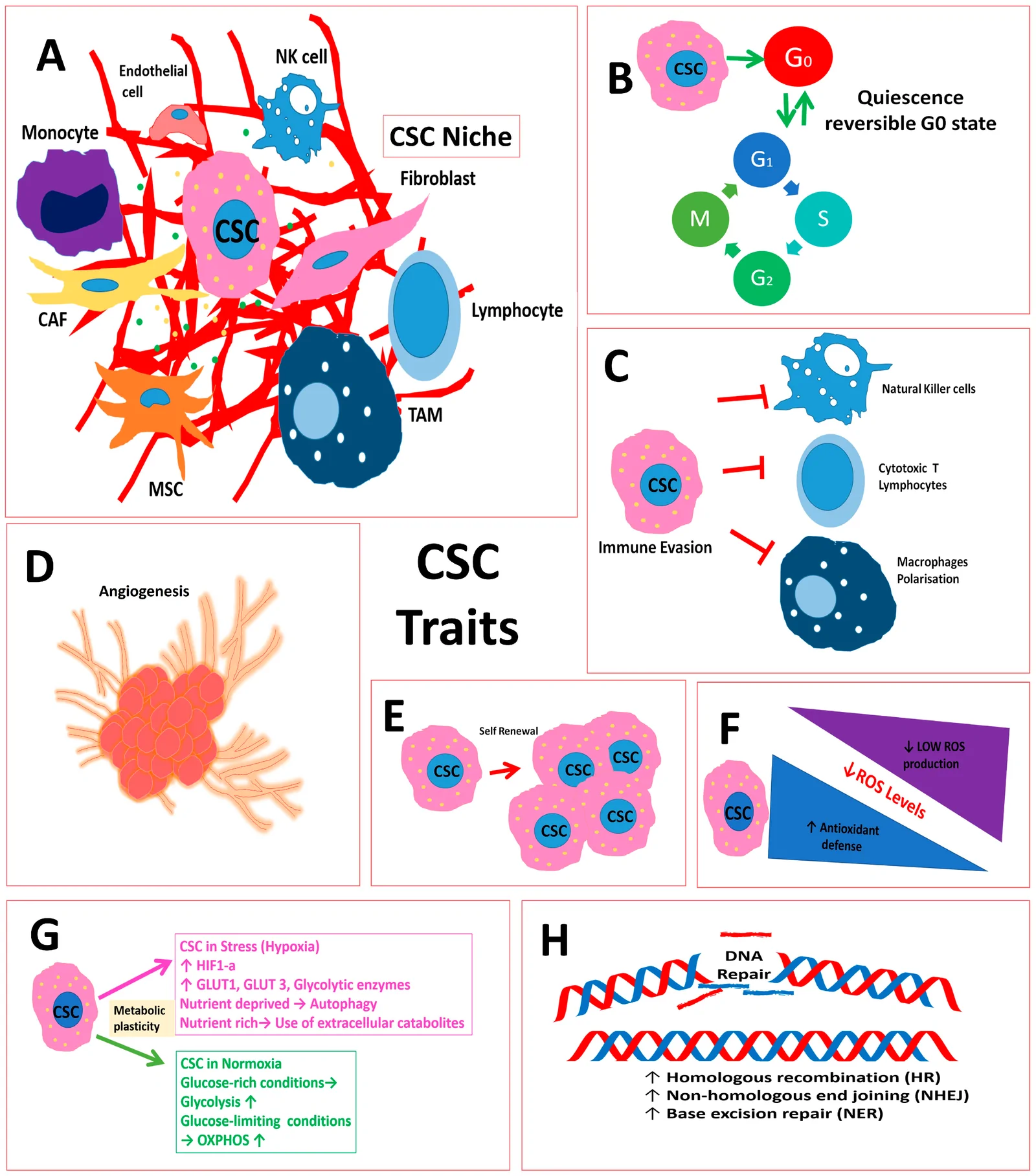
**Cancer stem cell (CSC) characteristics.** (**A**) CSC niche, cancer stem cells (CSCs) inhabit a specialized microenvironment composed of monocytes, endothelial cells, natural killer (NK) cells, fibroblasts, lymphocytes, cancer-associated fibroblasts (CAFs), mesenchymal stem cells (MSCs), and tumour-associated macrophages (TAMs), where reciprocal signalling sustains survival, proliferation, and therapy resistance. (**B**) Cell cycle and quiescence, CSCs can enter a reversible quiescent state (G_0_) to evade cytotoxic therapies, while retaining the ability to progress through G_1_, S, G_2_, and M phases for controlled proliferation. (**C**) Immune evasion, CSCs escape immune surveillance by inhibiting NK cell cytotoxicity, suppressing cytotoxic T lymphocyte activity, and polarizing macrophages toward tumour-promoting phenotypes. (**D**) Angiogenesis, CSCs promote neovascularization by releasing pro-angiogenic factors that drive endothelial cell proliferation and vessel formation, thereby supporting tumour growth and metastasis. (**E**) Self-renewal, CSCs sustain their population through symmetric and asymmetric divisions, generating heterogeneous progeny that fuel tumour progression. (**F**) Oxidative stress regulation, CSCs maintain low reactive oxygen species (ROS) levels via enhanced antioxidant defences, protecting against oxidative damage and contributing to therapy resistance. (**G**) Metabolic plasticity, CSCs adapt their metabolism by upregulating hypoxia-inducible factor 1-alpha (HIF1-α), glucose transporters (GLUT1/GLUT3), and glycolytic enzymes under hypoxia, engaging autophagy when nutrient-deprived, and switching between glycolysis and oxidative phosphorylation (OXPHOS) in normoxia depending on glucose availability. (**H**) DNA repair capacity, Cancer stem cells (CSCs) exhibit amplified DNA-damage response pathways that enhance survival under genotoxic stress. Elevated activity of homologous recombination, non-homologous end joining, and base excision repair enables efficient repair of double and single strand breaks. This heightened repair capacity reduces mutational burden, prevents apoptosis, and contributes to therapy resistance in CSCs.

Cancer remains a leading cause of morbidity and mortality worldwide [1]. Despite the availability of advanced therapeutics, cancer recurrence is the foremost concern against which biomedical science is still fighting [2]. The idea that cancer arises from cancer stem cells (CSCs) has its roots back in the mid-1800s but was finally established around 50 years ago through studies on teratocarcinomas [3]. The CSCs, also known as tumour-initiating cells (TICs), are a small population of cells within the tumour bulk possessing characteristics analogous to normal stem cells such as clonogenicity, self-renewability, heterogeneity and the ability to differentiate along multiple cell lineages (Figure 1) [4]. Owing to these characteristics, CSCs are increasingly recognized as potential initiating factors in certain cancers [4,5,6,7,8], and as key drivers of cancer progression [9], metastasis [10], therapy resistance [11], and relapse through their persistence and ability to reinitiate tumour growth [12,13].

Several studies have reported the presence of a proportion of cancer stem cells (CSCs) within the tumour bulk as a prognostic marker to predict patient survival and recovery in cancers, such as lung, ovarian, breast, thyroid, urothelial, head and neck (HN), glioma, hepatocellular, and gastric carcinomas [14,15,16,17]. In a comprehensive review, Lathia and colleagues studied across multiple malignancies and demonstrated that the expression of CSC markers including CD133, CD44, ALDH1 is consistently associated with poor overall survival, reduced progression-free survival, and adverse clinical outcomes, thereby underscoring the clinical impact of CSC biology. This review highlights how the latest CSC research can be applied by oncologists to tailor personalized treatment regimens for patients at different stages of cancer to support disease-free survival.

## 2. CSCs Implicated at Diagnostic Stage of Cancer

Cancer stem cells (CSCs) may be present in the primary tumour or may emerge later under the influence of complex microenvironmental factors [17,18]. Estimating CSC load and defining their molecular signatures in tumour tissue or circulation has been emphasized in the literature as a valuable approach for predicting disease prognosis and therapeutic response. Nevertheless, despite strong experimental evidence supporting the prognostic relevance of CSC quantification, these assessments are not yet incorporated into routine clinical diagnostics. This limitation arises from CSC heterogeneity, their transcriptional and signalling overlap with normal stem cells, and the cancer type-specific expression of biomarkers listed in Table 1, which collectively explain the absence of universal CSC markers [19]. Furthermore, CSCs constitute only a small fraction of the tumour mass, ranging from 0.01 to 2% depending on the malignancy [20], which complicates their isolation and accurate estimation in bulk tumours.

The identification of cancer stem cell (CSC) biomarkers remains highly context-dependent, with significant variability across tumour types. For example, CD133 is widely used as a CSC marker in glioblastoma and colorectal cancer, yet its expression is inconsistent in hepatocellular carcinoma and ovarian cancer, limiting its universal applicability [21]. Similarly, ALDH1 activity has been validated as a stemness marker in breast and ovarian CSCs, but its prognostic value varies depending on tumour subtype and microenvironmental context [22,23]. In haematological malignancies, CSCs are often defined by combinations of markers such as CD34+CD38− in acute myeloid leukaemia, but these phenotypes are not conserved across solid tumours [24,25]. This variability underscores the absence of universal CSC biomarkers and highlights the need for tumour-specific validation. Future research should focus on integrating transcriptomic and epigenomic profiling (e.g., scRNA-seq and ATAC-seq) to identify context-specific CSC signatures, while clarifying the limitations of current markers to enhance their translational value in clinical settings.

**Figure 2:**
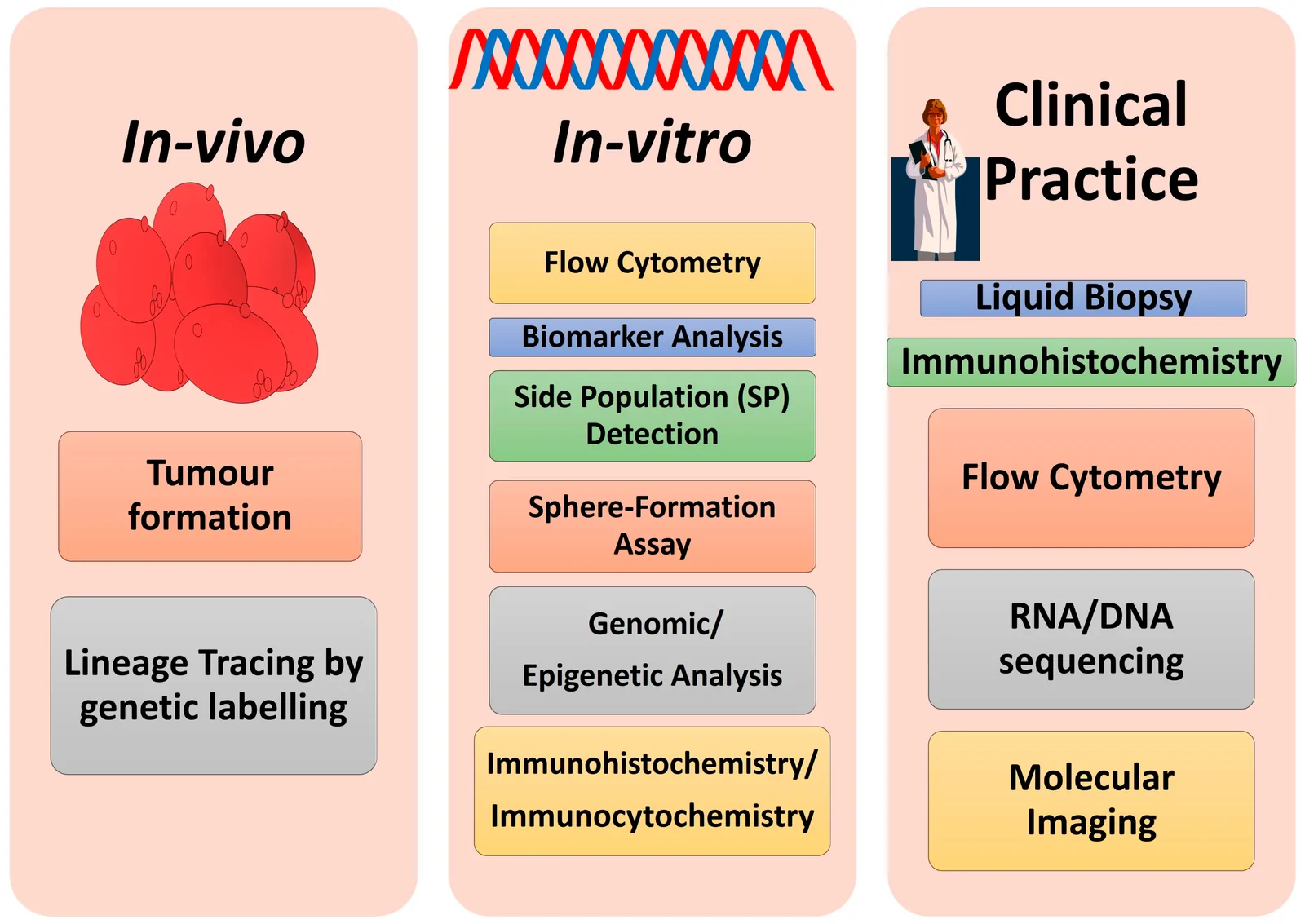
**Experimental and clinical approaches for CSC identification.** This figure compares methodologies used to identify and characterize cancer stem cells (CSCs) across three domains: in vivo, in vitro, and clinical practice. In vivo approaches include tumour formation assays and lineage tracing by genetic labelling; for example, lineage tracing enables researchers to follow genetically marked cells within animal models, thereby confirming stem-like properties such as self-renewal and differentiation in a physiological context. In vitro approaches encompass flow cytometry, biomarker analysis, side population (SP) detection, sphere-formation assays, genomic/epigenetic analysis, and immunohistochemistry/immunocytochemistry; for instance, the sphere-formation assay is widely used to assess the self-renewal capacity of CSCs under non-adherent culture conditions, providing functional evidence of stemness. Clinical practice approaches include liquid biopsy, immunohistochemistry, flow cytometry, RNA/DNA sequencing, and molecular imaging; for example, liquid biopsy allows detection of circulating tumour cells and CSC-associated markers in patient blood samples, offering a minimally invasive tool for monitoring disease progression and therapeutic response.

Numerous techniques have been developed to identify, separate, or quantify CSCs, as summarized in Table 2 and Figure 2; however, only a few have been adopted in routine diagnostic practice. Several methods have been validated in murine models or applied in cell culture systems [26], and intensive research is ongoing to refine these approaches for eventual integration into clinical workflows.

**Table 1:** List of CSC biomarkers in different cancer types.

Cancer Type	CSC Marker	References
Lung	CD133^+^, CD44^+^, ABCG2, ALDH, CD87^+^, CD90^+^	[13]
Melanoma	ABCB5^+^, CD20^+^	[27,28,29]
Ovarian	ALDH^+^ and CD133^+^ CD44^+^CD117^+^	[23,30]
Pancreatic	CD133^+^, CD44^+^, *α*2*β*1, ABCG2^+^, ALDH^+^ CD24^+^ESA^+^ NCAM, CD34, Thy-1, c-Kit, Flt-3	[13,31]
Ewing’s	CD133^+^	[32]
Breast	CD133^+^, CD44^+^, CD24^−^, EpCAM^+^, ALDH-1	[33]
Thyroid	CD44, CD166, and ALDHA1	[34]
AML	CD34^+^, CD38^−^ CD123^+^	[35]
B-ALL	CD34^+^CD10^−^/CD34^+^CD19 CD34^+^CD38^−^CD19^+^	[36]
T-ALL	CD34^+^CD4^−^/CD34^+^CD7^−^	[37]
Urothelial	CD14, CD44, CD90, and 67LR	[38]
HNSCC	SSEA-1^+^, CD44^+^, CD133^+^	[13]
Glioma	CD133, CD90, CD44, L1CAM, A2B5, and GPD1, B23, LGR5, CD133, Nestin, ABCG2	[39]
Prostate	CD133^+^, CD44^+^, *α*2*β*1, ABCG2, ALDH	[13]
Hepatocellular	CD133^+^, CD44^+^, CD49f^+^, CD90^+^, ALDH, ABCG2, CD24^+^, ESA	[40]
Gastric	CD133^+^, CD44^+^, CD24^+^	[13]
Colon	CD133^+^, CD44^+^, CD24^+^, CD166^+^, EpCAM^+^, ALDH, ESA	[13]
Multiple Myeloma	CD34^−^CD138^−^	[41]
CNS	CD133^+^	[42]

**Table 2:** Methods for Estimation of Cancer Stem Cell (CSC) Load.

Category	Method	Description	References
In vitro	Sphere-formation assay	Examines the ability of CSCs to self-renew and proliferate when maintained under non-adherent culture conditions.	[43]
	Aldefluor assay	Detects CSCs based on aldehyde dehydrogenase (ALDH) activity.	[22]
	Flow cytometry using surface markers	Identifies CSCs using specific markers like CD44^+^/CD24^−^, CD133, etc.	[44]
	Side population (SP) analysis	Uses Hoechst dye efflux to identify CSCs with high drug efflux capacity.	[45]
	Organoid culture	3D culture systems to grow CSCs and study their functional properties.	[46]
	Label-retaining methods (lipophilic dyes)	fluorescent and lipophilic dyes such as PKH26 and PKH6, BrDU and Carboxyfluorescein-succinimidyl ester (CFSE). CSCs being quiescent, retain these labelling dyes	[47,48,49,50]
	reactive oxygen species (ROS) levels	CSCs have low ROS levels, therefore based on the intensity of fluorescent stains for ROS, such as DCF-DA and dihydroethidium, CSCs can be detected	[26]
	CSCs exhibit higher mitochondrial membrane potential (Δψm)	Δψm heterogeneity based on variable fluorescence intensities of Rhodamine-123 (Rh123). Tetramethylrhodamine methyl ester (TMRM), and JC-1 dyes in CSCs	[26,51,52,53]
	Reporter system	Employs fluorescent proteins under EMT-inducer gene promoters to identify and isolate viable positive cells.	[54,55]
	Autofluorescence	Method based on intrinsic autofluorescence properties to isolate a subpopulation of glioma stem cells.	[56]
In vivo	Tumorigenicity in immunodeficient mice	Tests CSCs by injecting them into mice and assessing tumour formation.	[57]
	Lineage-tracing experiments	Tracks the origin and development of cells from single CSCs using genetic labelling.	[58]
	Zebrafish xenograft models	Studies CSC behaviour and drug responses in vivo using zebrafish as hosts.	[59,60,61]
Clinical	Circulating tumour cell (CTC) analysis	Detects CSCs in blood using surface markers and molecular characterization.	[62]
	Liquid biopsy	Uses blood-derived exosomes and cfDNA to detect CSC-specific markers.	[63,64,65]
	Immunohisto-chemistry (IHC)	Uses specific biomarkers that identify CSCs based on their unique properties of self-renewal, differentiation, and tumorigenicity.	[22]
	RNA sequencing	Profiles CSC-specific gene expression patterns in clinical samples.	[66]
	Molecular imaging	Magnetic resonance imaging (MRI) and positron emission tomography (PET) play major roles, while emerging techniques such as optical imaging are under development.	[67,68,69,70]

### 2.1. Artificial Intelligence (AI)

Artificial intelligence (AI), particularly deep learning, has become a powerful tool in biomedical sciences for automating complex image recognition, though its clinical use in oncology remains limited [71]. AI has already shown success in cancer diagnostics through histopathology, radiology, and multi-omics integration [72], but its application to cancer stem cell (CSC) detection is still at an early stage. CSCs present unique challenges because they share features with normal stem cells, lack universal markers, and exhibit heterogeneous phenotypes and metabolic states [18]. These complexities limit the availability of annotated datasets for robust AI training. To address this, generative approaches such as Conditional Generative Adversarial Networks (CGANs) for image-to-image translation have been proposed to improve CSC recognition in cultured cells, offering a way to overcome data scarcity [73]. In a recent study, Zhang and colleagues employed deep learning approaches using CGANs combined with Convolutional Neural Networks to analyse phase-contrast images of cancer stem cells (CSCs). By training the model on carefully selected high-accuracy datasets, the authors demonstrated that AI-managed image analysis could reliably distinguish CSCs from non-stem tumour cells within heterogeneous populations. This methodology allowed for precise delineation of CSC proportions in bulk tumour histological sections, highlighting the potential of AI-driven computational pathology to enhance quantification of CSCs and support translational oncology research [74].

Since CSC markers differ across tumour types, integrating AI-based image analysis with tumour-specific biomarker panels and stemness signatures may provide more reliable estimates of CSC populations within the bulk tumour [18]. This strategy could support personalized therapies by mapping CSC burden and predicting therapeutic resistance. Multimodal AI approaches that combine histopathology with genomics, transcriptomics, proteomics, and spatial omics are particularly promising for capturing CSC plasticity and microenvironmental influences [72]. Although clinical translation remains limited, outcome-driven validation and explainable AI frameworks are expected to accelerate adoption, positioning AI as a future cornerstone in CSC diagnostics and precision oncology [75]. Current innovations include image-based deep learning using CSCs and vision transformers to capture tumour architecture, stromal context, hypoxia, and EMT-related morphologies linked to stemness [76,77]; self-supervised learning to reduce reliance on exhaustive annotations [77]; generative models such as cGANs for dataset augmentation [73]; and deep learning applied to single-cell RNA sequencing and spatial transcriptomics to identify CSC trajectories and therapy-induced stemness [77].

### 2.2. Clinical Recommendations

Multiple studies have linked higher CSC load and gene signature to poor prognosis of cancer and higher mortality [14,78,79,80,81]. Therefore, it is prudent to identify the unique signature of CSCs and estimate CSC load at the diagnostic stage and when designing the post-surgical treatment regimen, in addition to standard investigations. The estimation of CSC load, genotype and reactivity can help oncologists to incorporate CSC targeting therapies in the treatment regimen, thereby ensuring elimination of CSCs and associated complexities such as cancer metastasis, recurrence and treatment failure [17]. Most of the reported methods to isolate and estimate CSCs from the tumour bulk have only been performed in research settings, and these techniques are not as yet in clinical practice. Major concerns are the extremely small CSC population, lack of availability of universal biomarkers and the fact that each tumour expresses specific biomarkers at variable levels. While biocompatible tracers combined with molecular imaging may eventually enable non-invasive identification of CSC signatures and load in vivo [82], equal emphasis should be placed on transcriptomic approaches such as bulk RNA-seq and single-cell RNA-seq (scRNA-seq). These technologies allow high-resolution mapping of CSC-associated gene signatures and quantification of CSC proportions within heterogeneous tumour samples, thereby complementing imaging strategies and offering a powerful avenue for translational integration into diagnostic and post-surgical treatment planning.

## 3. CSC Implications in Therapeutic Stage and Cancer Recurrence

The main therapeutic strategies to treat cancer involve surgery, chemo- and radiotherapies, either separately or in combination, immunotherapies and targeted therapies. CSCs have several innate physiognomies to combat common therapeutic strategies employed during cancer treatment, thereby leading to therapy resistance, relapse and metastatic spread [83,84,85]. Every treatment has its own detriments: for example, radiotherapy as well as chemotherapy can produce free radicals that can damage DNA, leading to oxidative stress [86]. These therapies efficiently target rapidly dividing cells, but CSCs usually escape for multiple reasons [13]. These are discussed below.

### 3.1. Metabolic Plasticity

CSCs can modify their metabolism and reset mitochondrial function in response to genetic, epigenetic, microenvironmental and pharmacological stresses to maintain their stemness characteristics [87]. Investigating CSC metabolic plasticity and the mechanisms that lead to therapeutic resistance remain critical research priorities. The major contributors involve altered levels of ROS, altered metabolic pathways and depolarized mitochondrial membranes in CSCs [88].

#### 3.1.1. Low ROS Levels and High Antioxidant Potential

Reactive oxygen species (ROS) are generated from oxygen through mitochondrial electron transport chain by-products and the activity of oxidases (e.g., NADPH oxidases, peroxidases) within diverse cellular compartments such as peroxisomes, plasma membranes and the endoplasmic reticulum [86,89]. These ROS mainly include hydrogen peroxide, superoxide and hydroxyl radicals, and act as secondary messengers by changing either their location, concentration or timing of action in different cell signalling cascades that protect CSCs from death, ensuring their survival [90]. Normal healthy cells maintain a fine equilibrium between ROS generation and their scavenging by antioxidant systems, whereas CSCs acclimatise to low-ROS environments by hyperactivating antioxidant systems (e.g., by increased production of glutathione (GSH) to enable their survival). As a result, they escape from common therapeutic regimens involving chemotherapy and radiotherapy that act either by increasing ROS levels or disabling antioxidant systems [91]. One example of hyperactive ROS scavenging systems is the increased aldehyde dehydrogenase (ALDH) expression in CSCs. ALDHs are important components of an antioxidant system comprising 19 members. These are involved in catalysing oxidation of aldehydes and are implicated in cellular detoxification by retinoic acid (RA)-based signalling that is protected from ROS. There are reports highlighting a strong link between increased ALDH levels, chemoresistance and the survival mechanisms active in CSCs [92].

In this complex survival process, transcription factors (TFs), such as forkhead homeobox type O (FOXO), hypoxia-inducible factors (HIFs), NF-κB, p53, and Nrf2, are activated predominantly through post-translational modifications and nuclear translocation events that enhance their transcriptional activity rather than simply elevated expression levels. For instance, Nrf2 is stabilised under oxidative stress and translocated to the nucleus, where it directly binds to antioxidant response elements (AREs) in the promoters of genes including *SODs*, *Prdxs*, *GPXs*, and *CATs*. In contrast, NF-κB and p53 are activated via phosphorylation and conformational changes, while FOXO and HIF respond to upstream signalling cascades (e.g., PI3K/AKT inhibition for FOXO, hypoxia-induced stabilisation for HIF), which increase their DNA-binding activity. Among these TFs, Nrf2 is the canonical factor that directly binds AREs, whereas NF-κB, p53, FOXO, and HIF regulate overlapping antioxidant and survival pathways through distinct promoter elements. Consequently, the active transcription of these antioxidant genes leads to overexpression of proteins such as Trx, Grx, Prdx, and CD13/aminopeptidase, resulting in a shift in the ROS balance. These findings have been reported in specific cancer contexts, for example, lung cancer and glioblastoma models, where Nrf2-ARE signalling drives antioxidant protein upregulation, and leukaemia stem cells, where NF-κB and p53 contribute to redox adaptation. Importantly, such mechanisms are not universal across all CSCs but vary by tumour type. Epigenetic regulation further fine-tunes CSC survival. Histone modifications (e.g., H3K4me3, H3K27ac), DNA methylation changes and repression of fructose-1,6-bisphosphatase 1 (FBPase1) alter promoter accessibility and glycolytic flux, lowering ROS levels and activating β-catenin signalling to sustain stemness in breast cancer [90,93,94,95]. In addition, CSCs with low ROS levels express high levels of the anti-apoptotic protein, BCL-2, further contributing to CSC survival. Several signalling pathways are also implicated in ROS regulation, including ATM, PTEN/PI3K/AKT/mTOR, activation of JAK/STAT, Notch and Wnt pathways [96,97]. Several miRNAs including let-7a, miR-34a, miR-21, miR-200, and miR-210 are also involved in modulating the balance of reactive oxygen species in CSCs [98].

As the internal and external ROS levels mainly damage DNA integrity, it is understandable that DNA repair proteins actively play a role in the survival of CSCs, and maintenance of a low ROS profile with the hyperactive ROS scavenging system to escape therapeutic interventions is key. This can be carried out either directly through their own redox activity and altered mitochondrial function, or indirectly by stabilising the ROS detoxifying system [93]. For example, apurinic/apyrimidinic endonuclease 1/redox effector factor-1 (Ape1/Ref-1 or Apex1) is highly expressed in CSCs from glioblastoma and pancreatic cancer models but not universally across all tumour types. Its C-terminal domain mediates DNA repair as part of the base excision repair (BER) pathway, while the N-terminal domain confers redox activity. Consequently, Ape1/Ref-1 regulates mitochondrial ROS, prevents apoptosis under ROS-inducing therapies, and enhances transcriptional activity of factors such as HIF-1α, p53 and NF-κB [99]. Furthermore, Apex1 promotes drug efflux in CSCs through transcriptional upregulation of ABCG2, thereby enabling these cells to evade the effects of therapeutic agents [99]. Another enzyme, ALKBH8 (Alkylation Repair Homolog 8), repairs DNA damage induced by alkylating agents and also maintains low ROS levels via NOX-1 (NADPH oxidase homolog-1), thereby contributing to anti-apoptotic signalling and survival [100].

#### 3.1.2. Altered Metabolic Pathways

CSCs utilise energy from a wide variety of substrates, such as glucose, pyruvate, lactate, hydroxybutyrate, glutamine, acetate, and fatty acids. This is influenced by multiple factors, including genetics, oxygenation, metabolic demands, pH and the surrounding tumour microenvironment (TME). There are conflicting reports about which are the main pathways employed by CSCs to produce energy, and these may be dependent on phenotype and TME [101]. For instance, CSCs residing in the perivascular niche benefit from ample oxygen availability and can utilize substrates such as fatty acids, amino acids, ketones, and lactate to produce ATP through oxidative phosphorylation (OXPHOS). In contrast, CSCs in poorly oxygenated environments predominantly depend on aerobic glycolysis, a pathway that is less efficient yet produces ATP more rapidly than OXPHOS [102]. CSCs have also been shown to be more dependent on glycolysis in specific cancers, including ovarian, nasopharyngeal, osteosarcoma, glioblastoma (GBM), and colon. Therefore, abnormally altered glycolytic intermediates or glycolysis products may serve as markers of CSC stemness and therapy resistance in these solid tumours. In addition, the glycolytic enzymes governing metabolic flexibility can be utilised as potential therapeutic targets to inhibit CSC stemness [102]. Taken together, there is no definitive agreement regarding the predominant energy substrates used by CSCs. Their metabolic phenotype appears flexible and context-dependent, involving shifts between glycolysis and glutamine utilization, or glycolysis and OXPHOS, while also incorporating secondary metabolic routes such as fatty acid oxidation, the pentose phosphate pathway, and glutaminolysis to meet their specific cellular requirements [101]. These metabolic shifts are aimed to maintain low ROS levels and high antioxidative capacity, which enable them to thrive in hostile conditions and replenish tumour bulk when required, finally leading to therapeutic failure. CSC fate, encompassing quiescence, proliferation, or differentiation, is largely dictated by metabolic plasticity under the regulatory influence of diverse factors present in the TME [103].

Another metabolic alteration in CSCs is the overexpression of enzymes associated with lipogenesis, especially cholesterol and fatty acid synthesis [104,105]. Increased production of cholesterol and lipids as well as increased uptake of exogenous lipids enables CSCs in breast and colorectal cancer CSC models to protect the cell membrane against peroxidation (mediated by chemotherapeutics and hypoxia) or the formation of scaffolds in the cell membrane to trap receptor molecules involved in CSC-related signalling [105,106]. In a study of colorectal cancer CSCs purified using CD133+ and ALDH+ markers, Begicevic et al. (2019) proposed that increased lipid uptake and hyperactive lipid metabolic pathways in CSCs culminate in overproduction of bioactive lipid signalling molecules, such as cholesterol, steroids, hormones and metabolites, inducing ABC transporters such as ABCG2 and ABCB1 (two widely accepted CSC markers) [106]. Lipid-mediated signalling in CSCs is also brought about by exosome overproduction in breast cancer models driven by increased uptake of lipids. However, precise details regarding the pathophysiology of enhanced exosome production and the contents of these lipid vesicles are unclear and require further investigation to identify possible therapeutic targets [106]. Altered lipid pathways have been demonstrated to convert non-CSCs into CSCs, and two enzymes have been shown to be very important in this regard: stearoyl-CoA desaturase 1 (SCD1) and β-hydroxy β-methylglutaryl-CoA reductase (HMGCR). In lung adenocarcinoma and ovarian cancer CSC models, stearoyl-CoA desaturase is involved in fatty acid synthesis [107], which enhances the production of lipid-modified Wnt proteins, causing activation of Wnt signalling and the resultant nuclear translocation of β-catenin/YAP/TAZ, leading to the activation of multiple transcriptional target genes, driving the conversion of cancer cells into CSCs. In these specific tumour contexts, CSCs were purified using CD133+ and ALDH+ markers, and functional assays confirmed stemness maintenance. The SCD1 also maintains stemness in CSCs via the NF-kB pathway, serving as a positive feedback loop for production of lipid desaturases [107]. The second enzyme, β-Hydroxy β-methylglutaryl-CoA reductase (HMGCR), regulates the conversion of HMG-CoA to mevalonate during cholesterol biosynthesis and is implicated in activation of Hippo and Wnt signalling, particularly in breast cancer and glioblastoma CSC models, where its activity has been linked to maintenance of CSC stemness [107,108].

#### 3.1.3. Depolarized Mitochondrial Membranes in CSCs

Mitochondrial functionality and integrity are important for cell proliferative and differentiation potential, and many mitochondrial features, such as reduced mitochondrial number, perinuclear arrangement, decreased ATP content, low oxygen/glucose consumption, low ROS, increased mitochondrial membrane potential (Δψm) and decreased mitochondrial DNA (mtDNA) quantity, have been proposed as indicators of CSC ‘‘stemness’’ [109,110]. Important pluripotency-related transcription factors, such as NANOG, SOX2 and OCT4, and EMT transcription factors TWIST1 and BMI1, also influence mitochondrial modifications in CSCs [111].

### 3.2. Therapeutic Targeting of Metabolic Plasticity

At present, metabolic events maintaining CSC stemness are poorly defined. Extensive research is required for their identification as these could represent potential therapeutic targets to eradicate CSCs, in addition to classical CSC markers. Lipid metabolism is an emerging viable therapeutic target, especially SCD1 and HMG-CoA reductase activity [106]. Important possible metabolic molecular targets are described in Table 3.

### 3.3. Quiescence

**Figure 3:**
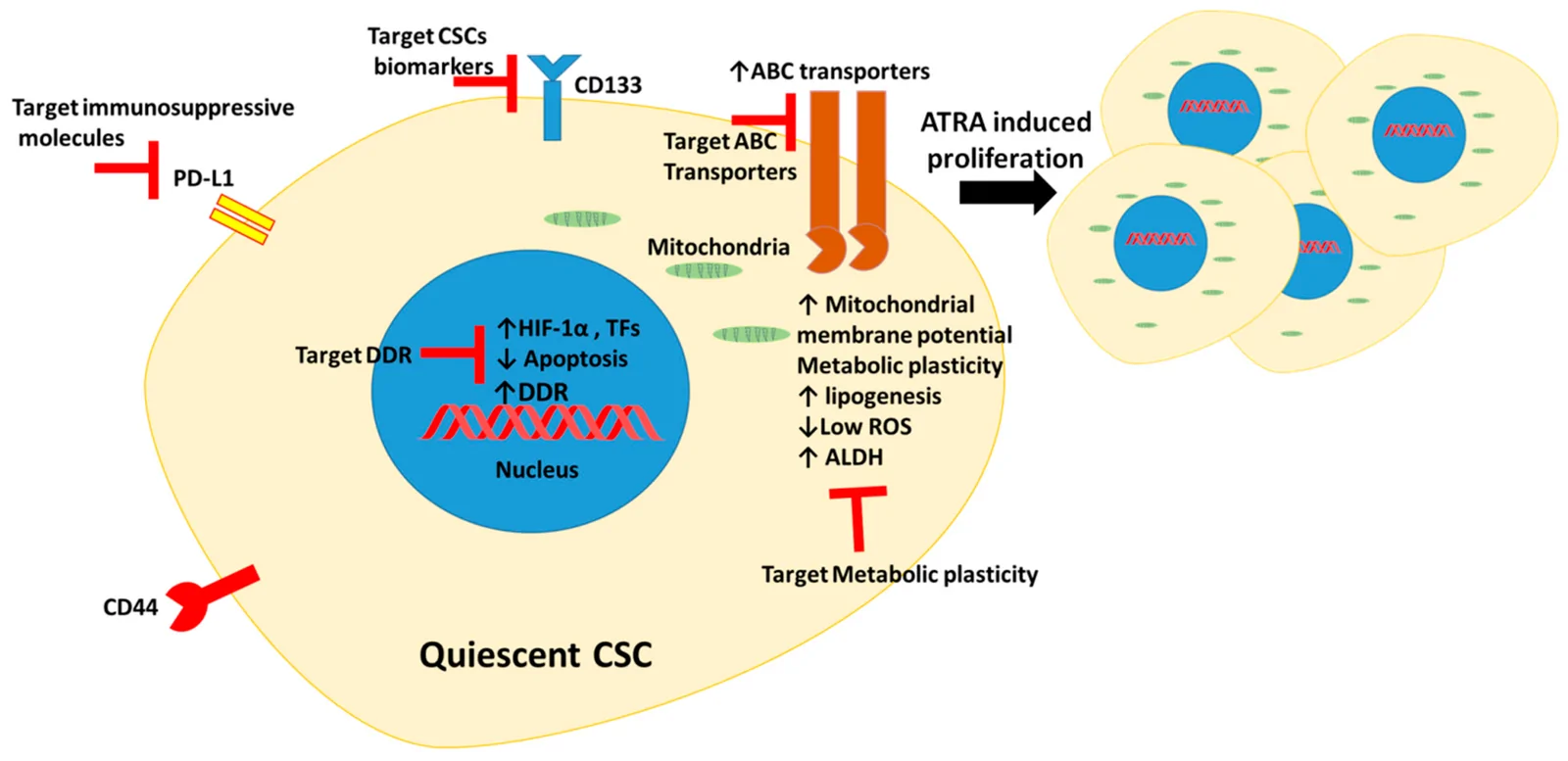
**Therapeutic targeting of CSCs.** CSCs could be eradicated by therapeutic targeting of specific biomarkers, angiogenesis, genetic and epigenetic factors, DNA repair system proteins, metabolic plasticity, apoptosis induction, inhibition of immunosuppressive molecules, NK and DC mediated immune activation, and targeting the TME and associated signalling molecules. Quiescent CSCs could be treated with all-trans retinoic acid (ATRA) to induce differentiation and proliferation, thereby sensitizing them to conventional chemo- and radiotherapies.

As a result of metabolic plasticity, CSCs can switch between quiescent or proliferative states under the influence of epigenetic and microenvironmental factors. CSCs can remain in a reversible G0 cell cycle phase (quiescence; Figure 3) for variable periods of time under unfavourable environmental conditions before re-entering the cycle and replenishing the tumour bulk (proliferative state), leading to therapeutic failure. As CSCs form a small portion of the main tumour, quiescent CSCs are in even smaller numbers; e.g., in breast cancer, around 5% of the total CSCs are estimated to be quiescent. These cells can be isolated on the basis of slow cycling, lacking proliferative markers, and low RNA content, and can be pulsed with labels such as the thymidine nucleoside analogue, BrdU [146].

To maintain a quiescent state, complex intrinsic and extrinsic pathways influenced by the TME, as well as genetic and epigenetic factors, are activated. Therefore, exploring these molecular mechanisms is crucial to identify therapeutically targetable molecules. Notable signalling pathways involved in regulating the quiescent state of CSCs include Notch, c-Myc/Wnt, and p38-MAPK [147]. The transcriptional regulation of genes controlling CSC quiescence is influenced by epigenetic mechanisms, including chromatin remodelling, DNA methylation and noncoding RNAs. Notably, breast CSCs demonstrate tighter heterochromatin formation via H4K20me3, whereas melanoma cells display elevated levels of the H3K4 demethylase JARID1B, highlighting distinct epigenetic strategies across tumour types [87]. Quiescent CSCs employ multiple mechanisms to escape immune detection, including diminished antigenicity and the expression of immune checkpoints such as PD-L1, which confer protection against T-cell-mediated cytotoxicity. Furthermore, angiogenic mediators like VEGFA and ANGPT2, together with cytokines such as IL-6, act in concert to promote immune evasion and ensure the persistence of quiescent CSCs [148].

### 3.4. Therapeutic Targeting of Quiescent CSCs

By remaining in a dormant state, quiescent CSCs drive resistance to chemotherapy in numerous cancer types, thereby sustaining tumour persistence and recurrence. The therapeutic targeting of quiescent CSCs is a new approach, and extensive basic and clinical research is required to identify and target relevant pathways. However, at this stage, the prospects are complex due to a lack of appropriate markers and the small population of quiescent CSCs. The TME is mainly responsible for maintaining the quiescent state of CSCs and protecting them from immune attack. In addition, it may promote the conversion of non-CSCs into CSCs. Therefore, targeting components of the TME is likely to be more effective in sensitising CSCs to chemotherapeutic drugs. One method is to force quiescent CSCs to differentiate (e.g., by treating them with all-trans retinoic acid (ATRA)), which first requires them to re-enter the cell cycle (Figure 3). The premise is that this will attenuate their resistance to conventional therapies and enhance elimination. However, CSC awakening may also create a risk of cancer progression by inducing a proliferative state [87,149]. Quiescent CSCs are in a low metabolic state; therefore, stimulating metabolic pathways such as glycolysis can re-sensitise the cells to chemotherapeutics, and CSCs can be forced into a differentiated state [149]. Cancer-associated fibroblasts (CAFs) play a role in chemoresistance through ECM remodelling and TME changes, thus supporting CSC survival. Therefore, forcing CAFs into a quiescent state has been suggested to increase the chemosensitivity of tumours, for example, by reducing vitamin D receptor overexpression driving tumorigenesis in CAFs by calcipotriol (a vitamin D analogue) in pancreatic tumours or ATRA in in vivo in melanoma [150,151]. Thus, induced quiescence of CAFs combined with conventional treatments could be a more potent therapeutic intervention [152]. Table 4 summarises strategies to combat quiescence in CSCs.

### 3.5. DNA Damage Response (DDR) System

There are conflicting reports about how DNA damage response (DDR) systems work in CSCs, and this has been attributed to tissue of origin, CSC heterogeneity and cell culture conditions used in different studies. In most CSC populations studied to date, DDR enzymes and proteins are upregulated, including ATM, ATR, CHK1, CHK2, PARP1, BRCA1 and RAD51 [160], in order to efficiently repair DNA; however, there are also conflicting reports of their downregulation in other CSCs including oesophageal CSCs [161]. Collectively, the published data suggest that upregulation of DDR systems in CSCs works efficiently to maintain their stemness, immune escape and survival. This raises the possibility that enzymes involved in the DDR of CSCs could be targeted therapeutically to eradicate CSCs [160]. In addition to DDR enzymes, CSCs tend to have a more open chromatin structure, although this requires further exploration to identify potential therapeutic targets [162].

Two key serine–threonine protein kinases in the DDR system that are upregulated in CSCs are ATM and the related ATR, in breast and cervical cancer [163], HNSCC [164] and glioblastoma CSCs [165]. These apical DDR kinases detect DNA damage and activate downstream target kinases, including CHK1 and CHK2. Compared to ATR, ATM has more widespread functions and it is active in chromatin reprogramming, oxidative stress, autophagy and cellular metabolism in normal and cancer cells [166]. During DNA damage, ATM phosphorylates KRAB-associated protein 1 (KAP1) at S824, which regulates chromatin relaxation and allows double-strand break (DSB) DNA repair [167]. In addition, it activates the pro-oncogenic kinases transforming growth factor-beta-activated kinase 1 (MAP3K7) and c-Jun N-terminal kinases (JNKs) and c-Jun/AP-1-dependent transcriptional activation of the PRNP gene that encodes the cellular prion protein (PrPC). Upregulation of this ATM/TAK1/PrPC axis mediates tumour cell radioresistance and is a known controller of CSC functions in neuroblastoma, breast, and colorectal cancer cell lines [99,168]. Similar to ATR, in triple-negative breast cancer models, oxidized ATM has been shown to modulate CSC populations independently of DNA damage pathways through metabolic reprogramming by activating glycolysis-related genes that include pyruvate kinase (PKM2), glucose transporter type 1 (GLUT1) and the tricarboxylic acid cycle (TCA)-associated gene pyruvate dehydrogenase (PDHα) through STAT5 (signal transducer and activator of transcription 5)/FOXP3 (forkhead box P3) signalling. Elevated acetyl-CoA production in this process induces epigenetic reprogramming via acetylation of defined lysine residues in histone H4, ultimately promoting CSC expansion [169]. The transcription factor ZEB1 is also activated by ATM in breast cancer and glioblastoma CSC models, where it induces EMT, tumour invasion, metastasis, and therapy resistance through stabilisation of CHK1 [170]. In addition, ATM upregulates autophagy-linked genes such as autophagy-related 4C (ATG4C) cysteine peptidase that sustain autophagic flux and mammosphere formation in breast cancer stem cell models [171,172], thus supporting CSC growth.

DDR proteins such as Telomeric Repeat Binding Factor-2 (TRF2; a component of the telomere-protecting shelterin complex) and Yamanaka transcription factor Krüppel-like factor 4 (KLF4) interact with pluripotent potential-related transcription factors such as Myc that further maintain CSC stemness and facilitate their survival in neuroblastoma, hepatocellular carcinoma and oral cancer models. These transcription factors also regulate DNA repair proteins in CSCs in a feedback loop manner [99]. Similarly, some DNA repair proteins act as transcriptional co-activators or co-repressors of CSC-related transcription factors. For example, BRCA1 regulates p53 transcription and acts as a co-repressor for Myc target genes. During DNA repair in breast and ovarian cancer models, BRCA1 is involved in multiple steps in the homologous recombination (HR) pathway, e.g., RAD51-dependent strand invasion and D-loop formation; therefore, loss-of-function mutations in BRCA1 increase cancer risk [173].

Certain DDR proteins play pivotal roles in sustaining CSC characteristics. For example, NBS1, an essential member of the MRN complex (MRE11, Rad50, and NBS1), stabilises HIF1α in response to radiation-induced DNA damage in breast, renal and oral cancer cell lines, thereby suggesting a potential role in the regulation of stemness-associated pathways [174]. Overexpression of NBS1 additionally promotes stemness and metastatic potential through activation of PI3K/AKT signalling in human cervical cancer cells and has been associated with pathways linked to stemness and tumour progression [99,175]. Likewise, high levels of MRE11, another MRN component, contribute to replication stress control, genomic stability, and survival of colorectal cancer stem cells through interplay with PARP1 and RAD51 [176]. Beyond these examples, DDR proteins such as DNA-PK and RAD51 participate in the regulation of innate immune signalling pathways. Emerging evidence indicates that DDR-dependent immune modulation can shape the tumour microenvironment and promote immune evasion, thereby creating conditions that favour CSC maintenance, persistence, and therapeutic resistance. DNA-PK has been implicated in immune regulatory signalling in glioblastoma models, whereas RAD51-mediated suppression of DNA damage-induced immune activation has been demonstrated in human fibrosarcoma cells and associated with immune dysregulation in oesophageal, breast, and colorectal cancers, potentially contributing to CSC survival and tumour recurrence [99,177,178,179].

### 3.6. Therapeutic Targeting of the DDR System in CSCs

The DDR system supports CSC stemness and survival by efficiently repairing DNA damage, regulating stemness-related transcription factors, detoxifying ROS and participating in CSC-related signalling [99]. Targeting DNA repair in CSCs presents a promising strategy to overcome treatment resistance, but its implementation is complicated by CSC heterogeneity, phenotypic plasticity, and their close resemblance to normal stem cells [161]. More focus should be given to identifying CSC-specific biomarkers and molecular signatures that distinguish them from normal stem cells. Recent RNA-seq and single-cell RNA-seq (scRNA-seq) studies in glioblastoma, breast cancer, and leukaemia have begun to address this by defining stemness-associated transcriptional programs and unique gene expression profiles that are enriched in CSC populations. Another new area of research is to study the mechanisms and basis of open chromatin in CSCs, referring to regions of accessible DNA that permit transcription factor binding and active gene expression. This can be determined using assays such as ATAC-seq or DNase I hypersensitivity profiling, which reveal chromatin accessibility landscapes. Such information is valuable for identifying CSC-specific regulatory elements and enhancer regions that drive stemness and therapy resistance, thereby providing novel therapeutic targets. Potential therapeutic targets of DDR system components in the eradication of CSCs are summarised in Table 5.

### 3.7. Apoptotic Inhibition in CSCs

In general, CSCs upregulate expression of anti-apoptotic proteins, enabling them to resist conventional chemo- and radiotherapies. The Bcl2 family proteins include anti-apoptotic Bcl-2, Mcl-1, Bcl-XL and pro-apoptotic Bak, Bax, Bid, Bim, Bik, Noxa, and Puma proteins. The inhibitor of apoptosis (IAP) family has many members, including IAP1, survivin, cIAP2, X-linked inhibitors of apoptosis (XIAP), NAIP, ML-IAP, and ILP-2. Apoptosis inhibition by these proteins is mediated by caspase-3, -7, and -9. Primary data from leukaemia and glioblastoma CSC models show dysregulated expression of Bcl2 family proteins with upregulation of IAPs and TRADD (the tumour necrosis factor receptor 1-associated death domain protein; a part of TNFR1 signalling that activates NF-κB in CSCs), leading to apoptosis inhibition, survival of CSCs and therapeutic resistance [190,191].

### 3.8. Therapeutic Targeting of CSC Apoptosis Inhibition Mechanisms

To re-sensitise CSCs to therapy, it is essential to elucidate aberrant apoptosis pathways. Bcl-2 inhibitors such as Venetoclax (ABT-199) have shown efficacy in CLL and sarcoma CSC models [192]. Other strategies include TRAIL receptor agonists such as dulanermin, which induces apoptosis via DR4/DR5 [193], and agonistic antibodies Mapatumumab [194] and Conatumumab [195] tested in xenograft and clinical models. Additionally, survivin-targeted dendritic cell vaccines have demonstrated immune activation and durable disease control in multiple myeloma patients [196]. These approaches highlight translational potential for overcoming CSC-mediated apoptosis resistance.

### 3.9. Tumour Microenvironment (TME) Components Support CSC and Treatment-Resistant Phenotypes

The tumour microenvironment (TME) functions as an interconnected ecosystem that sustains cancer stem cells (CSCs) and drives therapy resistance. Hypoxia, angiogenesis, cancer-associated fibroblasts (CAFs), tumour-associated macrophages (TAMs), myeloid-derived suppressor cells (MDSCs), cytokines, extracellular matrix (ECM) remodelling, and immune evasion mechanisms do not act in isolation; rather, they converge to reinforce CSC stemness, protect CSCs from cytotoxic therapies, and suppress anti-tumour immunity. For example, hypoxia stabilises HIF-1α to enhance stemness, angiogenesis supplies nutrients and immunosuppressive signals, CAFs remodel the ECM to sustain CSC niches, while TAMs and MDSCs secrete cytokines that dampen immune responses. CSCs themselves actively modulate dendritic cells and NK cells, inhibit cytolytic granules, and express immunosuppressive molecules to maintain survival. Recognizing these interdependencies highlights why therapeutic strategies must target both CSCs and their supportive microenvironment. We will briefly explain each component of the tumour microenvironment (TME) that supports CSC survival and treatment-resistant phenotypes in the following subsections.

#### 3.9.1. Hypoxia

Hypoxia occurs in solid tumours when the proliferation rate of cells surpasses the rate of neovascularisation. The result of an oxygen and nutrient-depleted environment is to activate a hypoxic transcriptional programme through hypoxia-inducible factors (HIFs) to adapt cancer cells to this hypoxic environment and support their continued survival. HIF1α is a key transcription factor that supports neovascularization, prevents cellular differentiation, controls cellular apoptosis, and activates DNA repair, all of which contribute to chemotherapy resistance [197]. HIF1α is implicated in drug resistance and an increase in the CSC population in several cancers, e.g., in colorectal cancer against 5-fluorouracil treatment via the CSN8/GLUT1-axis; and in hepatocellular carcinoma (HCC) cells against sorafenib resistance via the HIF1α/USP22/TP53-axis [87]. HIF1α also increases the CSC population in breast cancer through ITGA6 [198]; in pancreatic cancer through forkhead box protein M1 (FOXM1) [199]; via miR-215 in colon cancer; and through signal transducer and activator of transcription 3 (STAT3) activation in glioma [87].

#### 3.9.2. Angiogenesis

Crosstalk between the TME vasculature and CSCs sustains CSC niches and contributes to poor prognosis. CSCs induce angiogenesis by secreting pro-angiogenic factors and promoting differentiation of vascular endothelial cells; in return, the vasculature provides nutrients to support CSC survival. Important molecules and pathways related to angiogenesis in the TME include VEGFR-2, STAT3, MAPK/CXCL8/CXCL1 signalling, TGF-β, FGF2, b-FGF, stromal cell-derived factor SDF-1 (CXCL12), and angiopoietin [200]. In glioblastoma CSC models, VEGF secretion has been shown to drive abnormal vascular proliferation and sustain perivascular niches [201,202]. In breast cancer CSCs, CXCL1/CXCL8 signalling enhances angiogenesis and correlates with poor patient survival [203]. In hepatocellular carcinoma, CSCs activate STAT3 and TGF-β pathways to promote endothelial progenitor cell recruitment and vascular remodelling [202]. CSCs themselves can also aggregate to form abnormal leaky vasculatures mimicking blood vessel networks, thereby supporting tumour growth requirements, in addition to recruiting endothelial progenitor cells [202].

#### 3.9.3. Cancer-Associated Fibroblasts

CAFs are the predominant source of the extracellular matrix (ECM) in tumours and are essential for maintaining CSC niches within the TME. Their effects are mediated through secretion of collagens, fibronectin, ECM-degrading proteases, growth factors, angiogenic mediators, chemokines, and pro-inflammatory cytokines. In hepatocellular carcinoma, CAF-driven activation of lysine demethylase 1 (LSD1) enhances CSC self-renewal capacity [204]. In breast and lung cancers, a distinct CAF subset characterized by CD10 and GPR77 expression has been shown to directly sustain CSC populations, leading to chemoresistance and decreased overall survival [204,205]. In cholangiocarcinoma, CAFs secrete TGF-β and VEGF to promote CSC expansion and angiogenesis [206]. In oral squamous cell carcinoma, CAF-derived CXCL2 and CCR7 signalling enhances CSC stemness and invasion [207]. In colorectal cancer, CAFs regulate CSCs via integrin-mediated pathways and osteopontin (SPP1) secretion [208,209]. In osteosarcoma, CAF-derived connective tissue growth factor (CTGF) has been implicated in CSC maintenance and tumour progression [210]. Collectively, these examples demonstrate that CAFs regulate CSC populations in a tumour-specific manner through epigenetic modulation, paracrine signalling, and ECM remodelling.

#### 3.9.4. Tumour-Associated Macrophages

Macrophages are broadly classified into two types: pro-inflammatory M1 and anti-inflammatory M2. CSCs in the TME recruit macrophages and induce their differentiation into the M2 phenotype by secreting macrophage-specific chemo-attractants, such as CXCL8, IL-10, CCL5, CCL3, CCL2, ICAM-1, VEGF-A, neurotensin and a secreted ECM protein, periostin [211]. M2 macrophages also support self-renewal and CSC stemness in multiple cancers, including ovarian, glioblastoma, liver, breast and lung, by activating multiple signalling mediators such as STAT3 and NF-κB, leading to production of cytokines and growth factors, such as TGF-β, IL-6, IL-1β, IL-10, IL-8, and IL-17 [212].

#### 3.9.5. Myeloid-Derived Suppressive Cells (MDSCs)

MDSCs are immature myeloid cells that originate from hematopoietic stem cells. Several phenotypes of MDSCs exist, but only two are found in the immunosuppressive TME: monocytic MDSCs and polymorphonuclear MDSCs. Important factors that regulate their recruitment in the TME are growth factors such as G-CSF, M-CSF, and GM-CSF; cytokines such as IL-1, IL-4, IL-6, IL-13, and TNF; and chemo-attractants including IL-8, CCL2, and CXCL12 [213]. Myeloid-derived suppressive cells can enhance CSC stemness and survival by facilitating immune evasion, and this has been reported in several cancers including ovarian, pancreatic and breast [214].

#### 3.9.6. Cytokines

TGF-β is an important cytokine implicated in many aspects of cellular and cancer biology. In particular, it plays important roles in immune evasion, EMT induction, and complex CSC-related signalling in different tumour types, such as breast, lung and prostate cancers [215]. High IL-4 and TGFβ expression can lead to activation of SMAD2/SMAD3-dependent signalling in CD44^+^/CD24^−^ breast CSCs and support immune evasion. This is promoted by the human Sca-1 homologs Ly6K and Ly6E, reputed to be stem cell biomarkers [216] and urokinase-type plasminogen activator receptor (uPAR) [217].

IL-6 is another cytokine that is important for CSC induction. An increase in its expression can induce DNA methyltransferase 1 (DNMT1) through JAK2/STAT3 signalling that inhibits cell cycle regulatory factors in lung CSCs [218]. Tissue-associated macrophages (TAMs) secrete IL6, which triggers STAT3-dependent signalling in breast CSCs to maintain stemness [219]. IL-6/STAT3 pathway upregulation in CD44^+^ prostate CSCs [220] and HNSCC can upregulate PD-L1 expression, which promotes immunosuppression [221]. The CCL20/CCR6 axis is also linked to immune evasion via NFκB/PKCζ and p38MAPK pathways in breast, hepatocellular, oesophageal and lung CSCs [222].

#### 3.9.7. Extracellular Matrix

The extracellular matrix (ECM), as a key constituent of the TME, plays a crucial role in increasing the CSC population. Primarily consisting of collagen, laminin, fibronectin, proteoglycans, and matricellular proteins, it supports tumour angiogenesis and provides stem cell niches [202]. Hyaluronic acid (HA) can bind to CD44 to form a CD44-Nanog complex that promotes CSC stemness, metastasis and therapeutic resistance [223]. Laminin-332 (laminin-5) is specifically reported to be the cause of sorafenib resistance in HCC via the α3β1/Ln-332 axis [224].

#### 3.9.8. Impaired Antigen Presentation of CSCs

During immune interactions with tumour cells, T-cell receptors (TCRs) on cytotoxic T lymphocytes (CTLs) interact with MHC-I antigens of tumour cells. However, in CSCs, antigens are not properly degraded by proteasomal mechanisms; consequently, MHC-1-mediated antigen presentation is impaired. Therefore, the entire process of antigen processing and presentation is compromised in CSCs, leading to a defective cytotoxic response by CTLs [212,225]. In a tissue culture setting, melanoma spheroid cells downregulated expression of MHC-1, leading to a decreased allogenic immune response [226]. Similarly, neuro-spheres from primary glioblastoma enriched with CSCs expressed low MHC-1, lacked MHC-II, and demonstrated reduced expression of antigen-processing components, including low-molecular-weight protein (LMP), transporter associated with antigen processing (TAP), and β2-microglobulin [212]. Certain CSCs, including melanoma stem cells, achieve immune evasion through reduced expression of cancer-associated antigens and diminished levels of MHC class I and II molecules. This downregulation prevents effective recognition and targeting by T cells [227].

#### 3.9.9. Cytolytic Granule Inhibition by CSCs

NK cells and CTLs kill target cells by releasing cytolytic granules containing perforin and granzymes. Perforin oligomers make the membrane of target cells porous and induce necrosis. These, together with the help of granzymes, induce target cell apoptosis by activating pro-caspases. Breast CSCs can inhibit these cytolytic molecules by secreting protease inhibitor 9 (PI9), which inhibits granzyme B. Similarly, mast cell-induced serglycin, which is a proteoglycan with affinity for granzyme B, is responsible for immune escape of glioma CSCs. Pancreatic CSCs may escape immune surveillance of NK cells by expressing calreticulin that inactivates perforin [228].

#### 3.9.10. Expression of Immunosuppressive Molecules in CSCs

CSCs from multiple tumour types, such as gastric, breast, and bladder cancers, have been reported to express co-inhibitory immune molecules such as Programmed Death-Ligand 1 (PD-L1) to suppress immune surveillance [229]. PD-L1 on CSCs binds to PD-1 on immune cells and downregulates their functions. Multiple factors are involved in PD-L1 expression in CSCs, e.g., Ly6K/E [216], proteasome activator subunit 3 (PSME3) in breast cancer cells [230], DNA methyltransferase β3 (DNMT3B) in ALDH1^+^ radioresistant CSCs of oral squamous cell carcinoma [231], and HMGA1 in CD133^+^/CD44^+^ colorectal CSCs [232]. In addition, the insulin/PI3K/AKT/mTOR axis, EGF, STAT3, and acetylcholine/CD133/PI3K/AKT are also related to PD-L1 overexpression in different CSCs [212,233,234]. Another immunosuppressive molecule secreted by CSCs of multiple myeloma, AML and HNSCC is CD200 that binds to CD200R on macrophages and dendritic cells. This interaction suppresses macrophage activation and dendritic cell maturation, thereby reducing pro-inflammatory cytokine release and antigen presentation, promoting an immunosuppressive tumour microenvironment. CSC stemness-related pathways such as BMI1 and sonic hedgehog implicated in chemo- and radiotherapy resistance are also regulated by CD200 [235,236].

#### 3.9.11. Modulation of Dendritic Cell Activity by CSCs

Dendritic cells (DCs) are antigen-presenting cells required to elicit an immune response, and they play a critical part in regulation of tumour immunity and in linking innate and adaptive immune responses. Immature DCs capture the tumour-specific antigens and express them on their surface so that they can be recognised by the immune cells. DCs are derived from hematopoietic DC progenitor cells and can be classified as Langerhans cells, conventional DCs (with two subsets—DC1 and DC2), plasmacytoid DCs and inflammatory DCs [237]. DCs have either anti-tumour or pro-tumour effects depending upon their phenotype. CSCs escape immune surveillance by inhibiting their recruitment in the TME and inducing a tolerogenic immunosuppressive phenotype that supports a pro-tumour role. This phenotypic change is executed by secretion of multiple factors, such as IL-4, IL-10, IL-13, and TGF-β, PD-L1, B7-H3, CXCL12, CD47, IDO1 and signal regulatory protein alpha (SIPRα) [238].

IL-12 secreted by DCs can activate NK cells to release interferon-gamma (IFN-γ), which is crucial for metastasis suppression alongside CD4 and CD8 T-cell-mediated immunity in melanoma, colorectal and breast cancer models. NK cells can also recruit DCs of the anti-tumour phenotype to the TME by secreting CCL5 and XCL1, thus suppressing cancer growth. This crosstalk between NK and DC cells can be utilised in developing DC vaccines to evade CSCs by pulsing NK and DCs with tumour-specific antigens [239].

#### 3.9.12. Modulation of NK Cell Activity by CSCs

Natural killer (NK) cells represent a pivotal arm of the innate immune system, specialized in recognizing and eliminating transformed or infected cells that exhibit diminished or absent major histocompatibility complex class I (MHC-I) expression. Under physiological conditions, MHC-I molecules engage inhibitory receptors (e.g., KIRs, NKG2A) on NK cells, thereby attenuating their cytotoxic potential. Consequently, tumour cells that evade recognition by MHC-restricted cytotoxic T lymphocytes remain vulnerable to NK cell-mediated lysis [240].

CSCs exhibit heterogeneous susceptibility to NK cell attack, broadly categorized into NK-sensitive and NK-resistant phenotypes. For instance, breast CSCs characterized by CD24+/CD44+, CD133+, and ALDH+ expression profiles demonstrate elevated levels of MHC-I polypeptide-related sequence A/B (MICA/MICB), which serve as ligands for the NK cell-activating receptor NKG2D. This molecular configuration renders these CSCs more susceptible to NK-mediated cytotoxicity [238]. Similarly, colorectal CSCs display reduced MHC-I expression while concomitantly upregulating CSC-associated markers (CD133, CD44, Lgr5, CD166) and ligands for NK-activating receptors such as NKp30 and NKp44, thereby enhancing NK recognition and cytotoxicity [238].

Conversely, certain CSC populations adopt immune evasion strategies that confer resistance to NK-mediated killing. Some breast CSCs, for example, downregulate NKG2D ligands including MICA/MICB and UL16-binding proteins (ULBPs), thereby impairing NK cell activation and conferring resistance to cytotoxicity [241]. Additionally, CSCs may modulate apoptotic signalling pathways by altering the balance of pro-apoptotic and anti-apoptotic regulators, effectively blocking death receptor-mediated lysis. Another prominent mechanism of immune escape involves the upregulation of non-classical MHC-I molecules such as HLA-G and HLA-E. These molecules interact with inhibitory NK receptors KIR2DL4 (binding HLA-G) and NKG2A (binding HLA-E) to transmit suppressive signals that attenuate NK effector functions. Colorectal CSCs exemplify this strategy, thereby evading NK cell surveillance and contributing to tumour persistence and progression [242].

#### 3.9.13. Neural Regulation of the Tumour Microenvironment and CSCs

Emerging evidence highlights the role of neural signals, including neurotransmitters, neurotrophic factors, and autonomic nervous system activity, in shaping the tumour microenvironment (TME) and influencing cancer stem cell (CSC) biology. Neurotransmitters such as norepinephrine and acetylcholine can modulate angiogenesis and metabolic adaptation, thereby sustaining CSC niches. Neurotrophic factors, including nerve growth factor (NGF) and brain-derived neurotrophic factor (BDNF), have been implicated in promoting CSC stemness and invasiveness through activation of PI3K/AKT and MAPK signalling pathways. Autonomic nervous signals, particularly sympathetic innervation, contribute to immunosuppression by altering cytokine production and impairing NK cell cytotoxicity, while parasympathetic signals can influence tumour progression via cholinergic pathways. These neural-related signals, thus, represent an additional layer of regulation that connects the TME with CSC-driven drug resistance phenotypes. Importantly, spatial omics and single-cell analysis approaches now allow mapping of nerve fibre distribution in relation to CSC niches, providing insights into how neural CSC interactions are spatially organized within tumours [243,244]. Such analyses may uncover novel therapeutic targets by identifying neural-CSC crosstalk mechanisms that sustain stemness, invasiveness, and immune escape.

### 3.10. Therapeutic Targeting of TME and Immune Evasion in CSCs

Given the ability of the tumour microenvironment (TME) to sustain and activate stem cell-like programs in cancer cells, therapeutic targeting of TME components represents a promising strategy to mitigate chemotherapy resistance. Approaches to targeting CSCs include interventions against CSC-associated antigens, signalling pathways, cytokines, and immune cells, as summarized in Table 6. For example, NK and dendritic cells pulsed with CSC-specific antigens can be employed both for direct CSC killing and for the development of antigen-specific vaccines, highlighting innovative immunotherapeutic avenues. Because angiogenesis plays a crucial role in maintaining an immunosuppressive TME and supporting CSC self-renewal, combining VEGF-inhibitory strategies with immunotherapy may provide enhanced efficacy. Importantly, these mechanisms are interconnected: cytokine production, immune modulation, and angiogenesis collectively reinforce CSC survival and therapy resistance. To overcome these challenges, more extensive research and novel strategies are required for CSC eradication. Future directions include dissecting cytokine networks, mapping epigenetic regulation of immune evasion, and integrating angiogenesis inhibitors with immunomodulatory therapies to dismantle CSC niches and improve patient outcomes.

CSCs upregulate immunosuppressive molecules such as PD-L1 and CD200, inhibit cytolytic granule release from NK cells, and impair dendritic cell (DC) maturation, thereby reducing antigen presentation and T-cell activation [245]. Tumour-associated macrophages (TAMs) further reinforce CSC survival by secreting IL-10, TGF-β, and VEGF, which promote angiogenesis and suppress cytotoxic immune responses [246]. NK cell activity against CSCs is frequently attenuated by CSC-derived ligands that downregulate activating receptors such as NKG2D [247]. These findings highlight why single-agent CSC-targeted therapies are often insufficient, as CSCs exploit the TME to establish redundant immune escape pathways. Combination strategies such as pairing checkpoint inhibitors with CSC-targeted vaccines, or integrating VEGF blockade with NK cell-based therapies offer a rational approach to dismantling CSC niches and overcoming resistance. Future research should systematically evaluate these interactions to design multimodal therapies that simultaneously target CSCs and their immunosuppressive microenvironment.

**Table 6:** Targets of TME and immune evasion in CSC biology.

CSC Associated Molecules	Drugs	Mechanism of Action (MOA)	References
HLA	FAKE-NK100 SMT-NK	HLA/NK cell killing	[238]
EpCAM	CARTEPC	T cell cytotoxicity	[238]
CD133	CARTEPC	T cell cytotoxicity	[238]
JAK1/2	AZD-1480	Inhibit JAK/STAT pathway	[248]
STAT3	Celecoxib Pyrimethamine Napabucasin (BBI-608)	NQO1bioactivator/STAT3//Nanog pathway	[249]
IL6	Tocilizumab Siltuximab	IL-6R monoclonal antibody	[250]
IL8	Reparixin	CXCR1 inhibition	[251]
NF-kB	Acalabrutinib	BTK inhibition	[252]
	Ibudilast MN-166	TLR4 antagonist	[253]
	LCL-161	Inhibition of c-IAP	[254]
TGFβ	Fresolimumab Galunisertib Lucanix M7824	TGFβ 1/2/3 neutralizing antibody TGFβR1 inhibition TGFβ2 inhibition TGFβ/PD-L1 ligand trap	[255]
Wnt/β catenin	Ipafricept (OMP-4F28)	FZD8 receptor decoy	[256]
	Vantictumab (OMP-18R5)	Monoclonal antibody against FZD receptors	[257]
	PRI-724	CBP/β catenin antagonist	[258]
	WNT974	PORCN inhibition	[259]
	Defactinib	FAK inhibitor/Wnt pathway	[260]
PI3K/Akt	Duvelisib (IPI-145, Copiktra)	Dual PI3K-δ and PI3K-γ inhibitor/PI3K/Akt pathway	[261]
NOTCH	Al-101 MK-0752	Gamma secretase/S3 cleavage inhibition	[262]
	Nirogacestat Enoticumab	Monoclonal antibody against DL4	[263]
Hedgehog	Glasdegib (PF-04449913)	SMO-antagonist//Hedgehog pathway	[264]
VEGF	Axitinib	VEGFR blockage	[265]
	Bevacizumab	Inhibition of binding of VEGF to VEGFR	[266]
TAMs	Zoledronate acid	Mevalonate pathway elimination	[267]
TAMs	BMS-813160	CCR2/5 and inhibition of macrophages recruitment	[268]
TAMs	BL-8040	CXCR4	[269]
TAMs	Pexidartinib	CSF-1R inhibition	[270]
TAMs	AMG820	Monoclonal antibody against CSF-1R	[271]
TAMs	ALX148	CD47/SIRPα blockage	[272]
TAMs	IBI322	CD47/PDL1 specific antibody	[273]
TAMs	Hu5F9-G4	Monoclonal antibody against CD47	[274]
Myeloid Suppressor Cells	INCB001158	Arginase inhibition in MDSCs	[275]
NK	Lirilumab	KR, blockage of inhibitory signal of NK cells	[276]
NKG2A	Monalizumab PH2201	NKG2A/Inhibition of immune checkpoint	[277]
Tregs	Ontak	CD25/apoptosis induction	[278]
CD8^+^/CD4^+^ T cell	DC vaccines	Induces tumour-associated or -specific antigens (TAAs or TSAs) TAA/TSA-specific CD8+/CD4+ T cell responses	[279]
Nanog	Amcasertib (BBI-503)	Serine–threonine kinase inhibitor/Nanog and other CSC pathways	[280]
CXCR4	Plerixafor (Mozobil)	CXCR4 antagonist/CSC microenvironment	[281]
CXCR1	Reparixin (repertaxin)	CXCR1 inhibitor/FAK/AKT/FOXO3A pathway	[282]
SMO/Hedgehog	Sonedegib (LDE225, Odomzo) Vismodegib (GDC-0449, Erivedge)	SMO-antagonist/Hedgehog pathway	[17]

## 4. Conclusions

Cancer stem cells (CSCs) exhibit remarkable plasticity, transitioning between quiescent, proliferative, and differentiated states in response to treatment stress. This adaptability, driven by metabolic reprogramming, antioxidant defences that maintain low reactive oxygen species (ROS), and enhanced DNA repair capacity, collectively promotes drug resistance and recurrence. For example, CSCs can enter a resting G_0_ phase to evade therapies targeting cycling cells, shift between glycolysis and oxidative phosphorylation to survive hypoxia or nutrient deprivation, and activate DNA repair pathways such as homologous recombination and non-homologous end joining to withstand genotoxic stress. These mechanisms explain CSC persistence despite therapy and underscore the importance of timing and combination strategies, where metabolic inhibitors, ROS modulators, and DNA repair blockers are integrated with conventional treatments to disrupt CSC survival.

To overcome CSC-related recurrence and therapeutic failure, future approaches must employ precision strategies that selectively target CSCs while sparing normal tissue. Advances in molecular diagnostics will enable tailored treatments, while the integration of CSC-targeted therapies with chemo- and radiotherapy can enhance efficacy and reduce relapse. Novel modalities such as CAR-T-cell therapy, cancer vaccines, nanotechnology-based drug delivery, and CRISPR-mediated gene editing provide promising avenues to eliminate CSCs by disrupting survival pathways and resistance mechanisms. In parallel, artificial intelligence and machine learning can refine therapeutic design by identifying CSC markers and predicting responses, leading to more personalized interventions. By combining these innovations with ongoing oncology research, future treatments will offer more durable solutions, overcoming therapy resistance and significantly improving patient outcomes.

## Figures and Tables

**Table 3:** Metabolic targets of CSCs.

Potential Metabolic Target in CSCs	Available Drug Options	Mechanism of Action (MOA)	References
**Targeting OXPHOS**			
Electron transport chain (ETC) and OXPHOS	Metformins, Phenformin, Rotenone	Inhibit electron transfer of Complex I in ETC	[112,113]
	NV-128	Inhibit electron transfer of Complex I and IV in ETC	[51]
	Diphenyleneiodonium chloride (DPI) and deferiprone (DFP)	Inhibit electron transfer of Complex I and II in ETC	[114,115]
	Antimycin A and Atovaquone	Inhibit electron transfer of Complex III in ETC	[116,117]
	Bedaquiline, and oligomycin	Inhibit electron transfer of Complex V in ETC	[118]
Mitochondrial biogenesis	Epigallocatechin-3-gallate (EGCG), Salinomycin,	Wnt/β-catenin pathway inhibition	[119,120]
	Quercetin, ABT-263	Regulating the expression of Bcl-2	[121,122]
	XCT-790,	Block ERRA/PGC-1α pathway	[123]
	Azithromycin, Doxycycline, Tigecycline Metformin	Impair mitochondrial biogenesis by blocking protein synthesis and anti-mitochondrial respiration	[112,124]
ZEB1	miR200	Downregulate ZEB1	[125]
HIF-1α pathway inhibition	Fenretinide	Rapid ROS generation	[126]
Selenocysteine residue in TrxR1	Shikonin	Break ROS balance by targeting the selenocysteine/Inhibitors of Thioredoxin reductase 1 inhibitor TrxR1	[74]
**Targeting Glycolysis**			
GLUT 1 and Glycolytic enzymes	WZB117 Fasentin Phloretin Silibinin 2DG	Glucose transport inhibitors	[127,128,129]
	Ionidamine	HK2 inhibitor	[130]
	Bromodomain and extra terminal motif (BET) proteins inhibitors	Downregulation of oncogenic MYC mediated signalling	[131]
**Targeting Glutaminolysis**			
GLS1	Zaprinast	Inhibit GLS1 Blocks glutamine derivatives influx into the TCA cycle.	[132]
	BPTES	Inhibit GLS1 Blocks glutamine derivatives influx into the TCA cycle.	[133]
	CP839	Inhibit GLS1 Blocks glutamine derivatives influx into the TCA cycle.	[134]
	6-diazo-5-oxo-L-norleucine (DON)	Inhibit GLS1 Blocks glutamine derivatives influx into the TCA cycle.	[135]
Inhibitors of GSH	Temozolomide	Inhibitors of GSH	[136]
Nrf2	Hinokitiol Taxol	Nuclear factor erythroid 2-related factor 2 (Nrf2) inhibitors	[137]
	Chestnut leaf extract	Activation of Nrf2 blocked	[138]
Myc	Bromodomain and extra terminal motif (BET) proteins inhibitors	Downregulation of oncogenic MYC mediated signalling	[139]
**Targeting Fatty Acid Metabolism**			
FASN	Cerulenin	Inhibit fatty acid synthase FASN	[140]
ACC	Soraphen	Inhibit acetyl-CoA carboxylase (ACC)	[141]
JAK	AZD1480	Inhibit JAK/STAT3 signalling	[142]
PPARγ	GW9662	Block PPARγ (peroxisome proliferator-activated receptor-γ) signalling	[143]
UFA	CAY10566 SC-26196	Inhibit conversion of SFAs (saturated fatty acids) to monounsaturated fatty acids MUFAs	[144]
SCD1	Curcumin	Inhibit stearoyl-CoA desaturase 1 SCD1	[145]

**Table 4:** Strategies to target quiescent CSCs.

Target	Mechanism	Reference
IFNα STAT1	Induce proliferative phase	[153]
ITGB1	Integrin β1 (ITGB1) activation with activation of FAK, Src, ERK1/2, and MLCK	[154]
	miRNA-27b-3p and miRNA-455-3p	[155]
Wnt signalling	Porcupine inhibitor LGK-974 with Smoi inhibitor Vismodegib	[156]
Src with ERK1/2	Src inhibitor AZD0530 with MEK1/2 inhibitor AZD6244	[157]
NOTCH1/Gamma secretase	Targeting the γ-secretase complex by Gamma secretase inhibitors GSIs blocking NOTCH1	[158]
Cancer associated fibroblasts (CAFs)	Calcipotriol (a vitamin D analogue) ATRA	[159]

**Table 5:** Drug-targetable DDR system proteins in CSCs.

Target	Drug	Mechanism	References
ATM	KU55933 CP466722 KU59403 KU60019	ATM inhibitor, inhibition of DNA DSB repair at G2/M checkpoint	[180,181]
ATM/ATR	Caffeine	Inhibit ATM/ATR	[181]
DNA-PK	NU7441	DNA-PK inhibitor	[182]
CHK1/CHK2	DBH PF-00477736 XL-844 UCN-01	CHK1/CHK2 inhibitor	[181,183]
CHK1	MK-8776/SCH900776 (SCH) LY2606368 SB218078 AR458323 CHIR-124 CCT244747 GNE-783 GNE-900 SAR-020106 AZD7762	CHK1 inhibitor	[181,184,185,186]
CHK2	CCT241533 PV1019	Inhibition of CHK2	[181]
	Combination of antidiabetic drug metformin and antibiotic salinomycin	Inhibition of CHK2 (T68) phosphorylation	[99]
RAD51	RI-1 Amuvatinib DIDS B02 IBR2 Halenaquinone Fatty acid nitroalkeneOA-NO2 CAM 833 RS-1 CYT-0851 3E10 autoantibody Fab-F2-iPTD antibody	RAD51 inhibitor	[187]
ATR	VE821 VE822 VX-970	ATR inhibitor	[188]
PARP1/ PARP2	Olaparib AZD2281 ABT-888 BSI-201 AG014699 CEP8983/9722 MK-4827 Inotak INO-1001	PARP1/ PARP2 inhibitor	[189]

## Data Availability

No new data were created or analysed in this study. Data sharing is not applicable to this article.
